# The Effect of Adding Physician Recommendation in Digitally-Enabled Outreach for COVID-19 Vaccination in Socially/Economically Disadvantaged Populations Subtitle: A Randomized Controlled Trial

**DOI:** 10.21203/rs.3.rs-3604972/v1

**Published:** 2023-12-11

**Authors:** Kamal Sumar, Lisa Blue, Gina Fatahi, Atiq Bhatti, Mehek Sumar, Stephanie Alvarez, Pedro Cons, Nathalie Valencia, Zachary Williams, Sairam Parthasarathy, Chyke A. Doubeni

**Affiliations:** Adelante Healthcare; Providertech LLC; Ohio State University; Mayo Clinic; University of California San Diego; Adelante Healthcare; Adelante Healthcare; Adelante Healthcare; Adelante Healthcare; University of Arizona; Ohio State University

## Abstract

**Introduction:**

People from backgrounds that are economically/socially disadvantaged experienced disproportionately high COVID-19 death rates and had lower vaccination rates. Effective outreach strategies for increasing vaccine uptake during the pandemic are not fully known. Among patients receiving care at a Federally Qualified Health Center, we tested whether community engaged digitally-enabled outreach increased COVID vaccine uptake.

**Study Design, Setting, and Participants:**

A 3-parallel-arm randomized controlled trial with a hybrid effectiveness-implementation design was conducted among patients ≥18 years old on study enrollment during 2021 with 1,650 assigned in 3:10:20 ratio; 2,328 were later selected for two subsequent implementation rounds.

**Interventions:**

From April 13 to June 10, 2021, patients were proactively sent a text-messaging invitation to make an appointment for vaccination as part of the routine practice (Arm 1, n=150) with added personalized clinician recommendation alone (Arm 2, n=500) or with an explicit nudge for answers to frequently asked questions (Arm 3, n=1,000). Further implementation used messaging addressing vaccine hesitancy (n=1,323) or adverse reactions to vaccines (n=1,005).

**Main Outcomes and Measures:**

The primary outcome was the completion of the first SARS-Cov-2 vaccine dose determined at 14, 30 and 90 days after outreach.

**Results:**

Of 1,650 patients in effectiveness Arms, 61% was female. Vaccination rates for Arms 1, 2, and 3, were 6% (n=9), 5.4% (n=27) and 3.3% (n=33) at 14 days, and 11.5% (n=17), 11.6% (n=58), and 8.5% (n=85) at 90 days, respectively, which were similar in pairwise comparisons. At 90 days, vaccination rates were similar across the two implementation rounds (3.9% vs. 3.6%) and were similar to the rate (3.3%) among patients who were not selected for intervention arms or implementation rounds (n=8,671).

**Conclusions:**

Digitally-enabled outreach that included SMS messaging outreach augmented with clinician recommendations did not improve COVID-19 vaccination rates.

**Trial Registration:**

This study is registered at ClinicalTrails.gov Identifier: NC-T04952376

## Introduction

Vaccination is essential for containing pandemics but effectiveness in populations depends on reach, acceptability, and uptake. This is particularly important when multiple doses are needed, as is the case with the ongoing severe acute respiratory syndrome coronavirus 2 (SARS-CoV-2) pandemic. Equitable access to coronavirus disease 2019 (COVID-19) COVID-19 vaccination is a national health priority. Effective and scalable strategies for promoting vaccine uptake among people from groups that are economically or socially disadvantaged are essential for addressing COVID health disparities. People from economically or socially disadvantaged groups have lower uptake despite incurring higher hospitalization and mortality rates.^1–3^ Primary care clinicians/providers (PCP) are viewed as trusted sources for health information with the potential to promote vaccination,^4–6^ and digital tools are increasingly used in healthcare and have been used extensively during the COVID-19 pandemic. Direct outreach through text-messaging (short message service [SMS]) is commonly used in healthcare for scheduling appointments or notifying patients of healthcare related issues,^7^ but has had mixed results for promoting vaccination even with added behavioral nudges.^8–11^ The effect of framing SMS outreach as a PCP recommendation for improving vaccination, particularly in settings with low levels of engagement,^10^ is unclear. Federally qualified health centers (FQHC) in the U.S. serve groups that are underserved with health services, including many receiving Medicaid, or in low-socioeconomic status or “essential” jobs.^12^ This study investigated whether among people receiving care at a FQHC in a city with high COVID rates, SMS messages that include PCP endorsement increased COVID-19 vaccination uptake.

## Methods

### Study Design

We performed a 3-parallel-arm randomized pragmatic trial to test the effectiveness of proactive population-based outreach. Later, two separate rounds of implementation with refined messaging were performed. The study was conducted as part of the Arizona Community Engagement Alliance (CEAL) Against COVID Disparities Community Task Force, which worked to address misinformation, increase trust in vaccination, and identify and address barriers to COVID-19 preventive services.^13^ Our approach adapted ongoing strategies at a FQHC that included a survey as part of the CEAL initiative that included items on perceptions about SARS-Cov-2 vaccination. We collaborated with the leadership of the FQHC, which participates in the Arizona CEAL Community Taskforce. The study was registered on clinicaltrials.gov (NCT04952376) and approved by the Mayo Clinic IRB (Protocol # 21-002939), which waived informed consent requirements.

### Study Population

**Figure 1:**
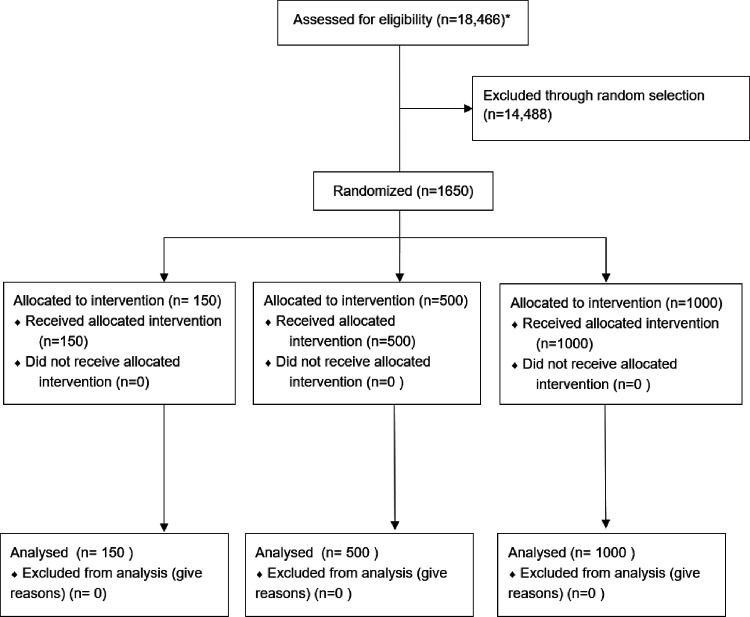
CONSORT Flow Diagram: *Note: We identified patients who during the 3/30/2020 to 3/29/2021 period were 18 years or older and had a medical visit. Visits were not limited by the type of clinician, clinic location, or vaccine status. We then restricted to those who had at least one visit with an MD or DO (FM or IM providers ONLY) during that period and did not have: · Upcoming scheduled vaccine appointment. · prior COVID vaccine dose documented in the electronic health record. Patients whose last visit was with an FNP or PA or whose visit occurred in two specific clinical locations (Wickenburg and Gila Bend) were excluded.

The study was conducted among people ≥18 years old who received care between April 13 to June 10, 2021 at Adelante Healthcare, which is a FQHC with community-based ambulatory primary care clinics in Phoenix, Arizona. FQHCs provide comprehensive primary care services regardless of insurance status or ability to pay.^12^ Eligible patients were identified using the electronic health record (EHR). We excluded people with documented vaccination; no medical visit within the previous year; and an upcoming appointment within 1 month (Figure 1). Among those eligible (n=18,466), we randomly selected and assigned 1,650 patients to one of 3 effectiveness arms in a 3:10:20 ratio per protocol. We subsequently selected an additional group of patients (n=2,328) for two implementation rounds of outreach.

### Interventions

The intervention was built on an existing population outreach program at Adelante FQHC that is implemented in partnership with Providertech, a healthcare technology company that operates an outreach platform with automated workflows. Because of observed trends of declining vaccination rates at Adelante and the region at the time the intervention was implemented and in consideration of CEAL’s interest in equitable access and to increase likelihood of engagement, we enabled the ability to respond via 2-way SMS to frequently asked questions (FAQs) for all participants. Thus, all participants, irrespective of the study arm, could use the two-way messaging feature by replying to the text message, and schedule an appointment or speak with clinical staff through a dedicated phone number provided through the Providertech platform (***Supplementary Figure S1***).

People assigned to Arm 1 (n=150) received the usual practice of proactive SMS outreach to the cell phone on file with a message that “it is your turn” to get vaccinated and an invitation to make a vaccination appointment. In Arm 2 (n=500), the message was personalized by including a statement from the PCP recommending vaccination (Table 1). We included a link in the messaging to FAQs for people assigned to Arms 2. Arm 3 (n=1,000) was similar to Arm 2 plus an explicit nudge to use an available 2-way dialogue option with the clinical team to obtain answers to FAQs.

FAQs were hosted on a cloned Adelante website that was created for the purposes of the study. Messages were developed through a community-engaged process and delivered in English and Spanish, based on patient preferred language on file, at preset times for each patient each week with up to 3 reminders.

The health center subsequently tested two implementation alternative messaging, which were sent separately on May 24, 2021, and 2 weeks later, based on responses to the preliminary survey. Those messaging outreach addressed potential COVID-19 vaccine hesitancy (Message A, n=1,323) or concerns about “side effects” or immune response to vaccination (Message B, n=1,005).

### Study Outcome

The primary outcome was the completion of the first COVID-19 vaccine dose as determined from EHR data ascertained at day 14, 30, and 90 after the outreach messaging. Outcome was also assessed in all 3 arms, the implementation groups, and among people not selected for effective or implementation.

### Statistical Analysis

Power calculations were based on data from other states in the CEAL program and had assumed a 42% vaccination rate in Arm 1 with a projected 20% higher rate in response to PCP recommendation and an additional 10% higher uptake in Arm 3 than Arm 2. Sample size was therefore estimated as 156 per arm for Arms 1 vs. 2 comparisons and 552 per arm for Arms 2 vs. 3 comparisons for 80% power at a 2-sided Bonferroni-corrected alpha of 0.017. We allowed for the potential for 20% ineligibility after randomization.

We used 2X2 contingency tables with the Chi-square test to perform pairwise comparisons of vaccination rates among the three Arms at each time point. Similar analyses were performed between implementation rounds (n=2,328) and also in people not included in intervention or implementation (n=8,671). Analyses were performed in STATA (StataCorp. 2019. *Stata Statistical Software: Release 16*. College Station, TX). Consolidated Standards of Reporting Clinical trial (CONSORT checklist) was followed to report the data.

## Results

A total of 18,466 were evaluated for the study, of whom 1,650 were randomly selected for the 3 Arms. Of those, 61.1% (n=1,008) were female, 64.4% (n=1,063) was White, 7.9% (n=130) Black or African American, and 47.4% (n=782) Hispanic/Latino that varied across Arms (Table 1).

Across the 3 arms, 88 patients reported prior vaccination. In an intention to vaccinate analysis, at 2 weeks of follow-up, the vaccination rates in Arms 1, 2, and 3 were 6% (n=9), 5.4% (n=27) and 3.3% (n=33), respectively. The difference was not statistically significant (P=0.78 to 0.05). Vaccination rates at 90 days (11.3% (n=17), 11.6% (n=58), and 8.5% (n=85), respectively, across Arms 1, 2, and 3) remained similar in pairwise comparisons (P=0.07 to 0.05).

Of the 2,328 who received the subsequent implementation round of outreach messaging, 650 (27.8%) responded back using the two-way platform. When the vaccination rates of the two implementation rounds were assessed at 90 days, both rounds had lower response rates than Arm 3 (p-value<0.01), and response rates to outreach addressing reactions or side effects or hesitancy were similar (Table 2). Among 8,671 patients at Adelante who were not selected for the interventions, vaccination rates were 0.44%, 1.3% and 3.3% at 14, 30, and 90 days, respectively. Sensitivity analyses that considered reported prior vaccinations did not change the findings (Table 2).

## Discussion

Due to its simplicity, low implementation cost, and scalability, digitally enabled outreach is an attractive approach for raising awareness on emerging public health issues and clinicians are considered trusted sources of information. In this study of a proactive population outreach intervention, adding a clinician recommendation did not improve the effectiveness of SMS messaging in improving COVID-19 vaccine uptake. Refining the strategy by offering to address concerns about vaccination also did not increase uptake. Our findings are in line with studies of proactive outreach in settings with low immunization rates that also reported similarly low uptake in response such as the 3.1–3.6% response rates that tested SMS +/− telephone calls.^10^ Our study was unique in the used of community-engaged approaches in low-resource settings. The underlying rates of vaccination during the study period were low at the FQHC, likely indicative of waning interest in the community over time in 2021. Consistent with our results, in previous studies of COVID and influenza vaccines that reported increased uptake with SMS behavioral nudges, the increases were modest.^8,9^ The ongoing use of SMS messaging in the population may have resulted in waning of effectiveness or reduced the potential effect of the additional nudges in our studies and may explain the lower uptake in Arm 3 and during implementation rounds. Thus, our results may be due to low engagement in vaccination related to sociodemographic factors, hesitancy, distrust, misinformation, and other barriers,^5^ which may not be easy to overcome with trusted messengers through digital media.

### Limitations

The benefit of digital tools during the pandemic is understudied in FQHCs. A limitation of SMS is the limited ability to deliver robust structured education or motivational counseling content that may be needed to address hesitancy. Our incorporation of interactive components did not improve effectiveness. There are other limitations of our study, including uncertainties about the completeness of capture of vaccinations received outside the health center, but findings were unchanged in sensitivity analyses. The digital divide may have also played a role in engagement in the intervention, but we were unable to assess the impact of digital health inequity on the response to SMS. Phone calls or SMS outreach for vaccinations are necessary for patients who have limited access to emails or internet services.^11^ We could not verify patient contact information before executing outreach. Community engagement is believed to promote vaccine uptake, and the CEAL programs had active community-wide vaccine education outreach during the time of the study.^13^ However. the effectiveness and reach of such strategies are unclear and could not be assessed in this study.

## Conclusions

Our results suggest that adding personalized physician recommendation to SMS messaging and a two-way interactive feature offering information about availability and addressing concerns is a feasible form of COVID vaccine outreach in under-resourced communities but did not increase uptake. Further research is needed on effective technology-enabled outreach strategies in populations receiving care in FQHCs.

## Figures and Tables

**Table 1.** Characteristics by Intervention Group Assignment of the Trial (N=1,650)

Characteristic	Arm 1 (n = 150) n (%)	Arm 2 (n=500) n (%)	Arm 3 (n=1000) n (%)
**Female**	92 (61.3)	323 (64.6)	593 (59.3)
**Age (years)**			
18 – 29	26 (17.4)	80 (16.0)	172 (17.2)
30 – 39	26 (17.3)	89 (17.8)	186 (18.6)
40 – 49	31 (20.7)	118 (23.6)	238 (23.8)
50 – 59	29 (19.3)	94 (18.8)	166 (16.6)
60 – 69	27 (16.9)	81 (16.2)	163 (16.3)
70+	11 (7.3)	27 (7.6)	53 (7.5)
**Race**			
White or European American	82 (54.7)	323 (64.6)	658 (65.8)
Asian American	3 (2.0)	15 (3.0)	38 (3.8)
Black or African American	12 (8.0)	40 (8.0)	78 (7.8)
American Indian/Alaskan Native	4 (2.7)	4 (0.8)	6 (0.6)
Native Hawaiian and other Pacific Islanders	0	2 (0.4)	9 (0.9)
More than one race	4 (2.7)	5 (1.0)	15 (1.5)
Unknown	45 (30.0)	111 (22.0)	196 (19.6)
**Ethnicity**			
Hispanic/Latino	82 (54.7)	249 (49.8)	451 (45.1)
Not Hispanic/Latino	62 (41.3)	231 (46.2)	522 (52.2)
Unknown	6 (4.0)	20 (4.0)	27 (2.7)

**Table 2.** Outcomes of study participants according to trial arms (n=1,650), implementation waves (N=2,328) and those not selected for implementation (n=8,671)

	Effectiveness trial			Implementation phase		
	Arm 1 N=150, n (%)	Arm 2 N=500, n (%)	Arm 3 N=1000, n (%)	Round 1 N=1,323, n (%)	Round 2 N=1,005, n (%)	Not Selected for Outreach N= 8,671, n (%)
14-day	9 (6.0)	27 (5.4)	33 (3.3) ^¶^	12 (0.9)	6 (0.6)	44 (0.5)
30-day	14 (9.3)	42 (8.4)	49 (4.9) *^¶^	22 (1.7)	14 (0.6)	89 (1.0) ‡
90-day	17 (11.3)	58 (11.6)	85 (8.5)	51 (3.9)	36 (3.6)	286 (3.3)

Significance:

1vs. 3;

* p<0.05

2 vs. 3:

¶ p<0.01

Baseline vs. Round 1:

‡ p<0.05

## Data Availability

The data that support the findings of this study are available from Adelante Healthcare, but restrictions apply to the availability of these data, which were used under license for the current study, and so are not publicly available. Data requests can be sent to Chyke A. Doubeni MD, MPH at chyke.doubeni@osumc.edu.
